# Defectors Cannot Be Detected during“Small Talk” with Strangers

**DOI:** 10.1371/journal.pone.0082531

**Published:** 2013-12-16

**Authors:** Joseph H. Manson, Matthew M. Gervais, Michelle A. Kline

**Affiliations:** Department of Anthropology, and Center for Behavior, Evolution and Culture, University of California Los Angeles, Los Angeles, California, United States of America; Hungarian Academy of Sciences, Hungary

## Abstract

To account for the widespread human tendency to cooperate in one-shot social dilemmas, some theorists have proposed that cooperators can be reliably detected based on ethological displays that are difficult to fake. Experimental findings have supported the view that cooperators can be distinguished from defectors based on “thin slices” of behavior, but the relevant cues have remained elusive, and the role of the judge's perspective remains unclear. In this study, we followed triadic conversations among unacquainted same-sex college students with unannounced dyadic one-shot prisoner's dilemmas, and asked participants to guess the PD decisions made toward them and among the other two participants. Two other sets of participants guessed the PD decisions after viewing videotape of the conversations, either with foreknowledge (informed), or without foreknowledge (naïve), of the post-conversation PD. Only naïve video viewers approached better-than-chance prediction accuracy, and they were significantly accurate at predicting the PD decisions of only opposite-sexed conversation participants. Four ethological displays recently proposed to cue defection in one-shot social dilemmas (arms crossed, lean back, hand touch, and face touch) failed to predict either actual defection or guesses of defection by any category of observer. Our results cast doubt on the role of “greenbeard” signals in the evolution of human prosociality, although they suggest that eavesdropping may be more informative about others' cooperative propensities than direct interaction.

## Introduction

Humans frequently cooperate with each other in one-shot anonymous economic games [1]–[3]. Despite considerable cross-cultural variation, the observed cooperation rate is much higher than predicted by an economic model based on pure self-interest, even when kin selection [4], [5] and reciprocal altruism [6] are taken into account. This poses an evolutionary puzzle: how did such cooperation evolve?

Two competing explanations are cultural group selection (e.g. [7]), and the prevalence, in ancestral environments, of a single interaction predicting future interactions with the same individual [8]. A third line of theorizing proposes that unrelated cooperators assort by self-identifying with voluntary signals [9]–[11]. Any such “greenbeard” signal is open to exploitation by deceptive defectors who falsely signal as if they are cooperators [12]. The Frank-Hirshleifer model attempts to solve this problem by proposing that moral emotions such as sympathy and gratitude motivate economically “irrational” generosity, while simultaneously generating ethological displays of intent to cooperate, that are reliable because they are difficult to fake. A functional link between generosity-motivating emotions and communicative signals of cooperation allows for reliable assortment among cooperators, while guarding against exploitation by defectors. One problem with this approach is that simply making signaling more costly or difficult for defectors would favor the eventual spread of any mutation allowing defectors to signal cooperation at the same low cost incurred by cooperators [13], [14]. Even commitment-related emotions such as guilt, operating in conjunction with predictive accuracy in a one-shot social dilemma, cannot prevent unconditional defectors from invading [15]. In contrast, an ongoing evolutionary arms race between deceptive signalers and skeptical signal receivers could generate a mix of frequent dyadic cooperation and rare exploitation [16].

Despite these theoretical problems, a growing body of research seems to support the Frank-Hirshleifer model in showing that people can judge others' propensity to defect in one-shot social dilemmas based on brief social interactions. Frank et al. [17] found that participants could predict others' choices in a Prisoner's Dilemma (PD) game at above-chance levels, after the three participants talked for 30 minutes—during which they could make unenforceable promises about game play. Brosig [18] replicated this result, even after excluding predictions made about participants who stated that they would defect. Reed et al. [19] also replicated this result. DeSteno et al. [20] found that strangers conversing face-to-face before playing an unannounced continuous PD game (dubbed the Give-Some Game) predicted each other's choices more accurately than strangers who interacted only via a web-based chat. Kikuchi et al. (1997, cited in [21]; T. Yamagishi, personal communication) found accurate PD play prediction following a neutral-topic discussion among strangers who did not know they would play a game.

Various sources of information may be reliably indexing future behavior in these studies [9]–[11]. Some evidence suggests that facial expressions may accurately signal cooperative intent. Reed et al. [19] found that individuals who smiled more (whether Duchenne or not) while promising cooperation in a one-shot PD were more likely to cooperate than those who displayed contempt. Studies measuring cooperation in a variety of ways have linked facial displays of emotion with cooperativeness or trustworthiness [22]–[25]. General emotional expressivity, regardless of positivity, may signal cooperativeness [26]. Schug et al. [27] found that individuals who made more generous offers in an Ultimatum game also showed more emotional expression in response to unfair Ultimatum game offers.

Particular gestures and postures may also reliably signal cooperative propensities. DeSteno et al. [20] found that individuals scoring higher on a unit-weighted aggregate of the frequencies of four behaviors (arms crossed, lean back, hand touch, and face touch) while conversing with a stranger transferred fewer monetary tokens in an unannounced post-conversation Give-Some game. A second experiment showed that a humanoid robot operated to produce these cues was expected, by human participants, to transfer fewer tokens in the Give-Some game, compared to the same robot when operated to produce other gestures.

The “thin slice” literature further suggests that immediately observable features of individuals may index cooperative dispositions. Research shows that “thin slices” of behavior can facilitate accurate judgments of stable individual propensities to cooperate [28]. The relevant cues may be stable physical traits. Facial masculinity in men is negatively associated with cooperativeness [29], [30], and men with lower second-to-fourth digit ratios (a proxy for prenatal testosterone exposure) are less trusting than those with higher 2D:4D ratios [31].

Most research in this area has been limited to individual-level attributes as cues to defection or exploitation. DeSteno et al. [20] draw attention to the importance of context-specific decisions to defect, and context-specific cues of impending defection. In fact, the majority of participants in social dilemma situations are conditional cooperators, not pure cooperators or pure defectors [32]–[35]. This suggests that actors' decisions are affected to some extent by perceived *dyad-specific* probabilities of cooperation, as well as the commonly studied individual-level and contextual variables [36]. Significantly, conditional behavior is not inconsistent with detection of future defection. “Thin slice” research has shown that people can quickly make accurate judgments about the quality of others' dyadic social relationships (e.g. therapist-client relationships) [37].

Defector detection may be thus facilitated by many sources of information both about stable behavioral dispositions and context-specific intentions. In addition, in naturalistic settings, the circumstances under which people assess each other's cooperative propensities are quite varied. This implies that a wide range of experimental designs is needed to illuminate the role of defector-detection in supporting the evolution of one-shot cooperation. For example, there is probably considerable variation in the extent to which defector detection is a salient goal while potential judges are processing information relevant to predicting future behavior. In most experimental defector-detection research, participants are informed about the nature of the judgment task they will complete, prior to the presentation of the stimuli (cf. Kikuchi et al. 1997, cited in [21]; [38]). In most experimental protocols, participants are informed of their impending social dilemma decision before conversing, and are (1) instructed not to discuss the game (e.g. [39]) or else (2) allowed to discuss and “disclose” their game-play decisions (e.g. [17]). The former must make for awkward social interactions, while the latter transforms the “defector-detection” challenge into the rather different task of “liar-detection” [40]. To our knowledge, only two pieces of published research have assessed the accuracy of defector-detection based on social interaction preceding an unannounced social dilemma: the DeSteno et al. [20] study described above, and the Kikuchi et al. (1997, cited in [21]), who found that only participants scoring high on general trust accurately predicted the PD decisions of co-participants.

Another issue that has received little attention in the defector-detection literature is that predictions about others' cooperation may differ as a function of whether the judge is a second party (recipient) or third party (observer), and whether the judge is present for the face-to-face interaction or sees a videotaped version of it. “Thin slice” research typically privileges experimental control over ecological validity by presenting participants with media-based stimuli [41], as does most research on defector-detection [22], [25], [28], [38], [42]–[44]. Only a few studies have asked participants to predict others' play following face-to-face interaction [17], [18], [20], [21].

At least three considerations suggest that patterns of prediction will differ between face-to-face and media-viewing contexts. First, it may be that only face-to-face interactions activate the neurophysiological and hormonal mechanisms underpinning cooperation or trust [45], [46]. It is unclear how this may affect the accuracy of predictions, one of the key empirical questions for theory on the evolution of cooperation. Second, actors may make predictions that reduce cognitive dissonance with respect to their own decisions; for instance, a defector may avoid feeling like a cheater by predicting that others will also defect. Finally, the cognitive demands of self-presentation to strangers might interfere with other cognitive tasks [47], including judgments of others' propensities to cooperate. This could decrease the accuracy of 2^nd^ party face-to-face predictions relative to those of a 3^rd^ party.

In the present study, we address these methodological issues by investigating how two experimental tools frequently used in the study of defector-detection—video-mediation for third party judges, and prior knowledge of an upcoming game—may affect the behavioral predictions that judges make about participant game play in a one-shot PD. We do this by forming conversational triads and comparing the predictions by four types of judges: First, the conversation participants (who had not been told about the PD before beginning the conversation) guessed their two co-participants' decisions toward themselves (2^nd^ party). Second, the conversation participants guessed their two co-participants' decisions toward each other (3^rd^ party insider). Third, a separate set of participants guessed the conversation participants' PD decisions after viewing a video of the conversation, without being told about the PD before viewing the video (naïve 3^rd^ party outsiders). Finally, another set of participants were told about the PD decision-guessing task before viewing conversation video (informed 3^rd^ party outsiders).

We address several specific empirical questions. First, do the four types of judges differ in the rates of baseline cooperation they predict? Second, are predictions concordant across judge types? Third, which, if any, of the four judge types can make accurate predictions? Fourth, do judges expect intra-individual consistency across decisions? And fifth, irrespective of accuracy, what cues or variables inform judges' predictions?

The analyses on defector detection presented here build on our findings regarding the actual determinants of our conversation participants' PD decisions [48]. We found two main effects: people were more likely to cooperate (1) if they grew up in a wealthier zip code and (2) towards more facially attractive co-participants. We also found two interaction effects with subclinical primary psychopathy (callous affect, interpersonal manipulation) as measured by the Levenson Self-Report Psychopathy Scale [49]: people higher in primary psychopathy were less likely to cooperate toward co-participants (1) who had interrupted them more frequently and (2) with whom they had discovered no common ground (e.g. shared acquaintance or academic major). We interpreted these results as supporting a view of subclinical primary psychopathy as a strategy of selective defection toward prospective social partners perceived to be of low value. One goal of the present paper is to determine whether the independent variables that affect an individual's actual PD decisions also affect observers' guesses of that individual's PD decisions. Another goal is to replicate DeSteno et al's [20] findings regarding ethological cues of untrustworthiness in an unannounced one-shot social dilemma following a conversation among strangers. In general, we found a lack of accurate defector detection and a lack of agreement among different guessers. These findings cast doubt on the role of defector detection in the evolution of human cooperation.

## Materials and Methods

### Participants

This study involved three distinct groups of participants. Conversation participants (*N* = 105) were recruited on a USA college campus [48]. The publicized study title was “Small Talk Among Strangers.” All participants were offered $10 USD compensation. Participants were scheduled in groups of three same-sexed individuals, and were screened upon arrival to make sure they had not met previously. The median participant age was 19 years.

Naïve third party outsiders (hereafter, naïve video viewers: *N* = 70, 49 female) and informed third party outsiders (hereafter, informed video viewers: *N* = 35, 28 female) were recruited from the same participant pool, during academic years following the completion of the conversation/PD trials. Naïve video viewers received course credit and a $6 payment, and could earn an additional $6 by making accurate predictions about gameplay (see below). Informed video viewers received course credit and a $3 payment, and could earn an additional $12 by making accurate predictions about gameplay. Third party participants were not asked their ages.

All procedures were approved by the UCLA Institutional Review Board (Approvals #G07-10-097-01 to -04; #G10-01-004-01; and #10-000371). Written informed consent was obtained from all participants in accordance with the terms of these approvals.

### Procedure

Conversation participants were informed that their conversation would be videotaped. Logistical and resource constraints limited the protocol to a single video camera, which directly faced one of the three participants. The identity of this participant was determined randomly. From the point of view of video viewers, the other two conversation participants were in profile. After 10 minutes of conversation, participants played an unannounced, computer-moderated one-shot PD toward each of the others. During the PD, they could “Keep” for themselves ( = defect) or “Transfer” to a recipient ( = cooperate) a $3 endowment provided by the experimenter; transferred funds were doubled and added to recipient payoffs. Unilateral defection yielded $9, mutual cooperation yielded $6, mutual defection yielded $3, and unilateral cooperation yielded $0. Participants then guessed how each co-participant played toward them (second party predictions) and toward one another (third party insider predictions). Each correct guess earned them $1. Prior to game play, we informed participants that one of them would not receive their earned payoff, but instead a randomly chosen, realistic payoff. This protected the confidentiality of participants' PD choices without eliminating their relevance to payoffs (see [17]). After the conversation, gameplay, and predictions, participants completed the Levenson Self-Report Psychopathy Scale (LSRP) [49] and reported their childhood zip code. All game play and questionnaire data were gathered using z-Tree Version 2.1 [50]. Finally, participants were photographed and given payoffs in sealed envelopes.

Coding of interruptions is described elsewhere [48]. For the entire 10-min duration of every video, a research assistant, ignorant of our hypotheses, coded every onset and offset of the four behaviors found by DeSteno et al. [20] to predict smaller transfers to co-participants in a Give-Some Game: *arms crossed*, *lean back*, *hand touch*, and *face touch*. To ensure comparability, we obtained a detailed coding protocol from D. DeSteno (personal communication). A second research assistant, also ignorant of our hypotheses, independently coded 6 randomly chosen conversations of the 35 (i.e. 18 of 105 participants) for these four behaviors.

JHM prepared video and still photographs for presentation to the naïve and informed video viewers, using SuperLab 4.0. Each naïve video viewer viewed one 10-minute conversation video. Video viewers first acknowledged they had never met the participants in the video. Prior to their viewing, we told naïve video viewers that the video would show a “conversation among three people meeting for the first time. After viewing the tape, you will be asked some questions about the behavior of these people.” After showing the video, we presented the PD instructions to the video viewer, who was asked to guess participant PD decisions (“Keep” or “Transfer”) in each direction for each dyad of conversation participants. This yielded six PD guesses per viewer. Each viewer watched only one conversation video, and each video received a total of two viewer ratings. The procedure was identical for the informed video viewers, except that, before showing them the video, we showed them the PD instructions and explained that they would be asked to guess game play following the video viewing. Informed viewers repeated this for a second video; naïve viewers moved on to rate 21 conversational participants for facial attractiveness on a six-point Likert scale. (They did not rate participants from the video they watched). Both naïve and informed video viewers, like conversation participants, were awarded $1 USD for each correct guess of a PD decision. See [48] for additional details.

### Data Analysis

See [48] for details of calculating attractiveness scores, psychopathy scores, childhood zip code median income, and interruption rates. Following DeSteno et al. ([20] and personal communication), we used the individual's mean frequency of onsets of the four putative cues of untrustworthiness (*arms crossed*, *lean back*, *hand touch*, and *face touch*) as an independent variable hypothesized to be inversely associated with probability of cooperating.

We examined frequencies of agreement among guessers, and guesser prediction accuracy, with respect to their deviations from chance frequencies based on the base rates of actual cooperation and predictions of cooperation (see [17]). Since each guesser evaluated multiple conversation participants, we examined inter-rater agreement and accuracy of game play predictions using log-linear (poisson regression) models rather than Kappa [51], in order to control for non-independence of ratings. In all cases, we present the conservative standard errors and confidence intervals based on data clustered by the individual guesser.

In relating our independent and dependent variables, we used bivariate and multivariate logistic regression models. Since each actor made multiple and therefore non-independent predictions, we calculated robust standard errors of the odds ratios, clustering by the identity of the individual making the predictions, before calculating confidence intervals and *P*-values. For all analyses involving PD decisions, cooperation was coded as 1, and defection as 0. All tests are 2-tailed. The data for this study can be accessed in the Dryad repository [52].

## Results

We obtained adequate inter-rater reliabilities of (1) the coding of interruptions between JHM and a research assistant, and (2) facial attractiveness ratings among participant raters (naïve video viewers) [48]. For the 18 participants whose frequencies of the four gestural/postural behaviors were coded by two research assistants, Cronbach's alpha between the two coders' mean values of the four behaviors was 0.98.

Coders were unable to reliably code frequencies of at least one of the 4 gestural/postural behaviors for 15 of the 105 (14.3%) conversation participants. Such cases included, for example, 11 individuals seated in the chair directly facing the camera whose *lean back* behavior could not be reliably coded. These 15 individuals were excluded from analyses of the gestures/postures.

Other missing data points included two conversation participants who declined to play the PD, one conversation participant who declined to guess her co-participants' PD decisions, and 4 naïve video viewers and one informed video viewer each who declined to guess one PD decision. Missing data points were excluded from analyses on a casewise basis.

### Base rates of predicted cooperation are generally inaccurate

**Figure 1:**
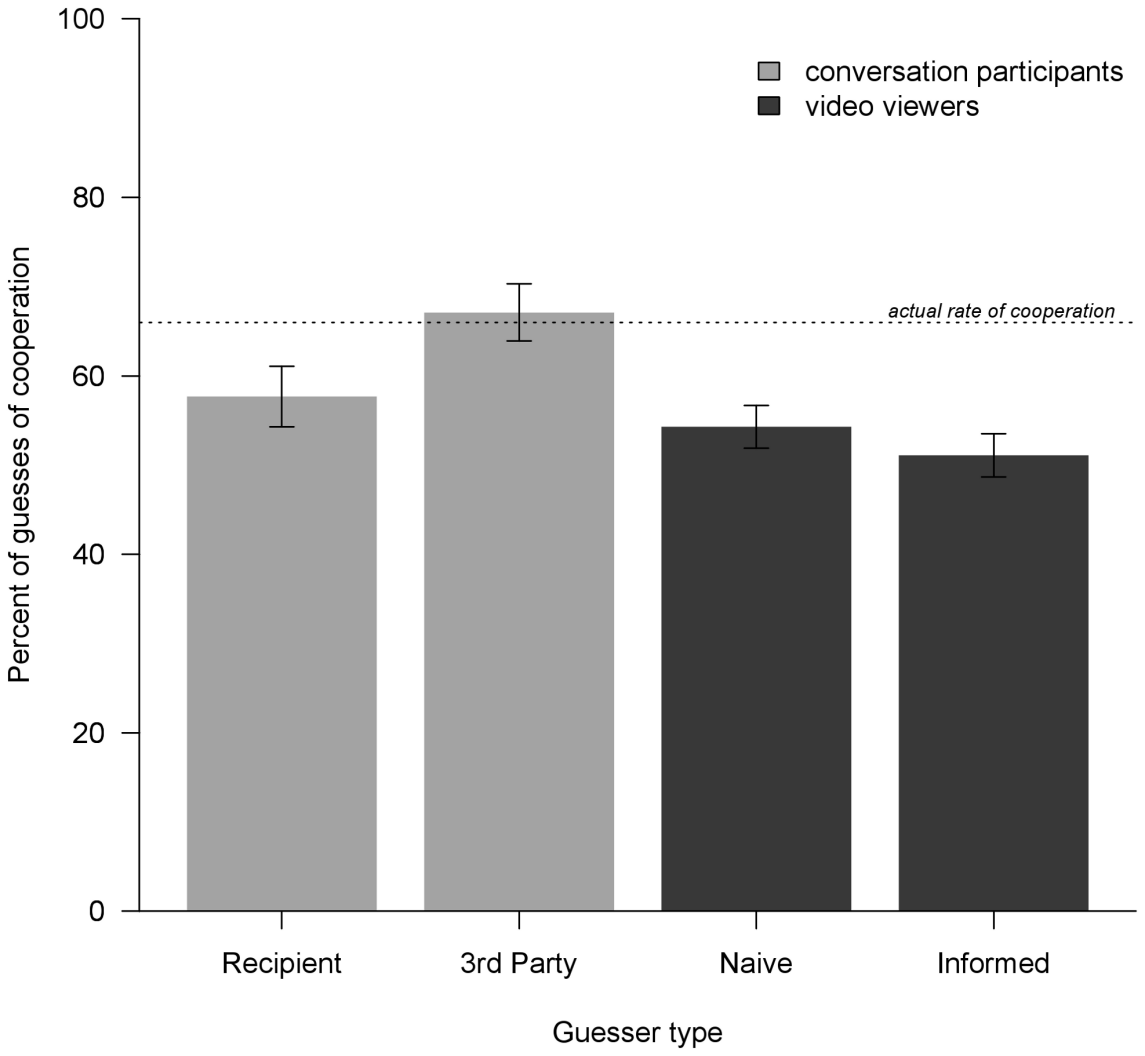
Percentages of guesses of cooperation, relative to actual cooperation rate, as a function of guesser type. Guesser types are Recipients of the PD decision (*N* = 208), Third party insiders (*N* = 210), Naïve video viewers (*N* = 416), and Informed video viewers (*N* = 419). Standard errors are indicated. Two-sample difference of proportion tests: Actual vs. Informed, *P*<0.001; Actual vs. Naive, *P*<0.01; Recipient vs. Third party, *P*<0.05; Third party vs. Naive, *P*<0.01; Third party vs. Informed, *P*<0.001. All other differences were non-significant.

In Gervais et al. [48], we report that 136/206 (66%) of actual PD decisions were to cooperate. Figure 1 compares this to the percentage of guesses of cooperation by the four guesser types: *recipient*'s guesses of *actor*'s decisions toward herself (2^nd^ party); *other*'s guesses of *actor*'s decisions toward *recipient* (3^rd^ party insider); naïve video viewer (3^rd^ party outsider) guesses; and informed video viewer (3^rd^ party outsider) guesses. Two-sample tests of proportions revealed that unlike conversation participants, video viewers (both naïve and informed) significantly underestimated the actual base rate of cooperation. Third party insiders, compared to all three other types of guessers, expected a significantly higher rate of cooperation that was not significantly different from the actual base rate.

### No agreement among participants' PD gameplay guesses

The six participants who guessed each PD decision did not agree with each other above chance levels. None of the three pairs of guessers (2^nd^ vs. 3^rd^ party insiders; two naïve video viewers; two informed video viewers) agreed at above chance levels (2^nd^–3^rd^ party insiders: δ±SE = −0.062±0.051, *P*>0.2; naïve video viewers: δ±SE = 0.007±0.067, *P*>0.9; informed video viewers: δ±SE = .009±0.097, *P*>0.9). Furthermore, for all PD decisions (*N* = 204) guessed at by all six participants, we calculated the observed aggregate frequencies (0–6) of guesses of cooperation, and compared these with the frequencies expected by chance based on the observed base rates of cooperation predictions made by the four categories of guessers. If guessers tended to agree, we would expect an overrepresentation of high (5–6) and low (0–1) counts. In fact, the observed counts of 6, 5, 4, 3, 2, 1 and 0 cooperation guesses (5, 32, 59, 54, 42, 10 and 3 respectively) were not significantly different from the expected counts of 6.1, 29.3, 58.4, 61.5, 36.1, 11.2 and 1.4 (χ^2^ = 4.01, df = 6, *P*>0.6).

### PD game guesses are generally inaccurate

**Figure 2:**
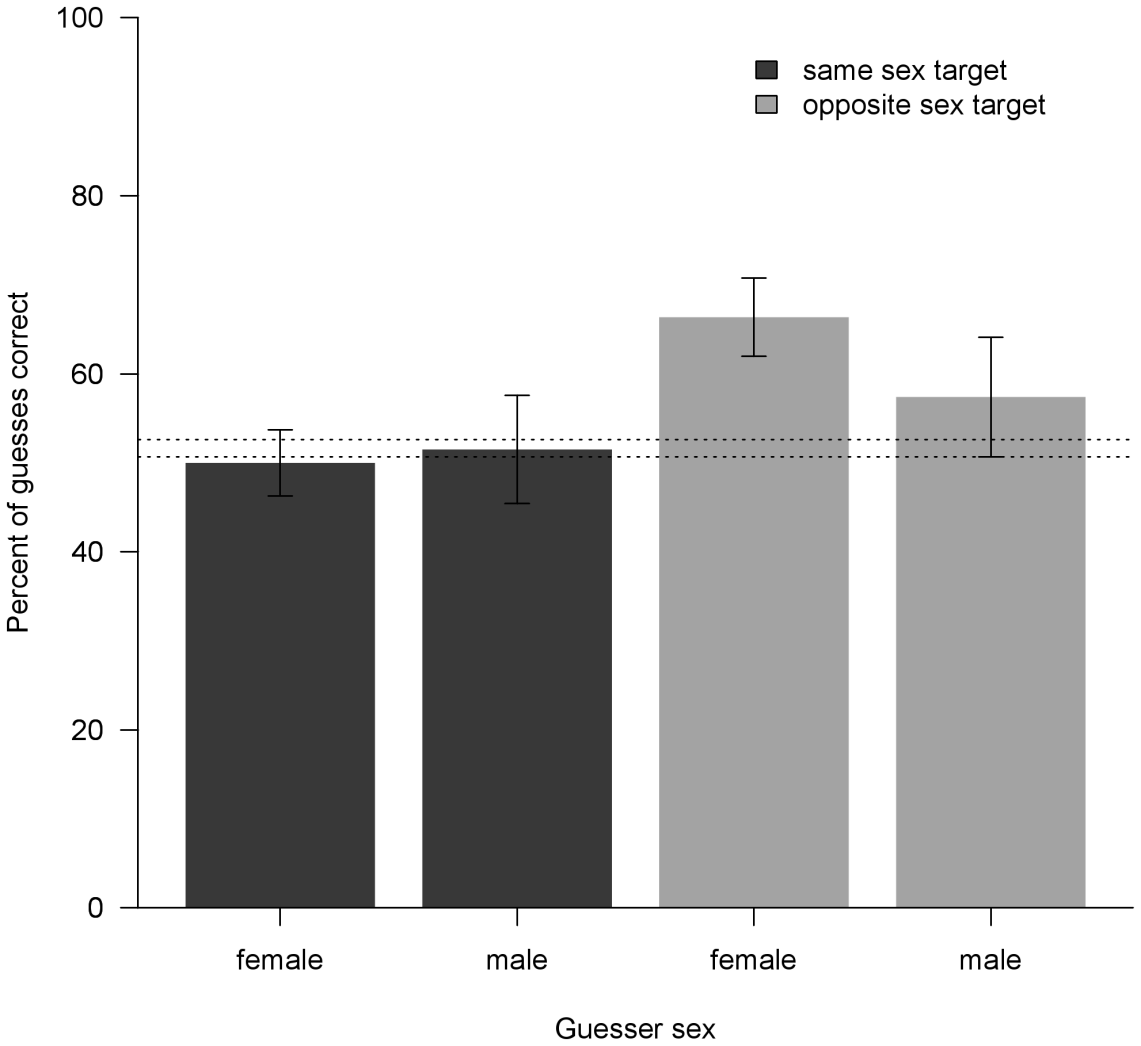
Accuracy of naïve video viewer PD guesses as a function of sexes of guesser and target. Dashed lines bracket the percentages of correct guesses expected under H_0_, which varied among guesser-target pairings as a function of the base rates of actual PD decisions and guesses. Standard errors are indicated. Guesser-target pairings are females guessing females' PD decisions (*N* = 176), males guessing males (*N* = 66), females guessing males (*N* = 113), and males guessing females (*N* = 54). In 7 cases, guesses could be not classified as correct or incorrect, because the target made no PD decision.

Table 1 shows descriptive statistics and analyses (poisson regression) of the accuracy of PD game guesses by the four classes of guessers: (1) 2^nd^ party (*recipient* guessing *actor*'s PD decision toward himself); 3^rd^ party insider (*other* guessing *actor*'s PD decision toward *recipient*); (3) naïve video viewer and (4) informed video viewer. Only naïve video viewers approached significant accuracy. Although we did not predict any sex effects on video viewers' prediction accuracy, post-hoc examination of our data revealed that this trend toward accurate prediction was driven entirely by the guesses of the 29 naïve video viewers who predicted the PD play of conversation participants of the opposite sex (δ±SE = 0.307±0.103, *P*<0.01), whereas the 41 naïve video viewers who predicted the play of same-sex conversation participants guessed at chance levels (δ±SE = 0.004±0.084, *P*>0.9) (Figure 2). Females predicting the play of males (*N* = 19) were especially accurate (δ±SE = 0.295±0.125, *P*<0.05), while males predicting the play of females (*N* = 10) were marginally accurate (δ±SE = 0.364±0.199, *P* = 0.067). For the informed video viewers, we did not find any sex interaction effects.

**Table 1:** Accuracy of guesses by four classes of guessers.

Guesser class	*N* (guesses)	Clusters (guessers)	Expected correct guesses	Observed correct guesses	δ^c^±SE	*P*
2^nd^ party^a^	206	103	108.1	109	0.00±0.05	>0.80
3^rd^ party insider^b^	206	103	116.5	112	0.00±0.06	>0.90
Naïve video viewer	409	70	210.4	228	0.12±0.07	0.06
Informed video viewer	411	35	206.6	213	0.00±0.07	>0.90

*recipient* guessing *actor*'s decision toward herself.^a^

b *other* guessing *actor*'s decision toward *recipient.*

c coefficient of delta in Poisson regression model.

Predictions of the PD decisions made by conversation participants facing the video camera (naïve video viewers: δ±SE = 0.057±0.063, *P*>0.3; informed video viewers: δ±SE = −0.006±0.086, *P*>0.9) were no more accurate than predictions of the decisions made by conversation participants who were not facing the camera (naïve video viewers: δ±SE = 0.074±0.073, *P*>0.3; informed video viewers: δ±SE = −0.009±0.106, *P*>0.9).

### Video viewers underestimate intra-individual consistency in PD play

Of the 105 conversation participants, 79 (75.2%) predicted that both their co-participants would make the same PD decision toward both that person's co-participants. In contrast, only 19/70 (27.1%) of naïve video viewers guessed that all three conversation participants would make the same PD decision toward both their co-participants. Five of 105 (4.8%) conversation participants predicted that both their co-participants made one decision to cooperate and one decision to defect, whereas 6/70 (8.6%) naïve video viewers predicted that all three conversation participants would make divergent decisions toward their two co-participants. Similarly, among the 35 informed video viewers, the mean (± SD) number of conversation participants (out of six) that were guessed to make consistent PD decisions toward both co-participants was 3.34±1.19. In fact, over 90% of conversation participants made the same PD decision toward both co-participants [48].

### Variables affecting guesses of PD decisions

Tables 2, 3, 4, 5, 6, 7, and 8 show the results of bivariate and multivariate logistic regressions in which the guesses (cooperate or defect) of each class of guesser is the dependent variable, and the independent variables are (1) *actors*' unit-weighted frequencies of the four behaviors *arms crossed*, *lean back*, *hand touch*, and *face touch* (henceforth, the DeSteno et al. [20] cues) and (2) those independent variables that constituted the most predictive model of actual PD play as reported by Gervais et al. [48]: *recipient*'s attractiveness, *actor*'s childhood zip code median income, *actor*'s primary psychopathy LSRP score, *recipient*'s rate of interrupting *actor*, *actor*-*recipient* common ground discovered during conversation, and the statistical interactions between *actor*'s primary psychopathy score and *recipient*'s rate of interrupting *actor*, and between *actor*'s primary psychopathy score and the discovery of *actor*-*recipient* common ground. The four multivariate models (Tables 6, 7, 8) vary with respect to their inclusion of (1) *recipient*'s PD decision toward *actor* (for the 2^nd^ party guesses) or *other*'s guess of *actor*'s PD decision toward him- or herself (for the 3^rd^ party insiders) and (2) the frequency of DeSteno et al. [20] cues displayed by *actor*.

**Table 2:** Bivariate logistic regressions predicting 2^nd^ party guesses (*recipient* guessing *actor*'s PD decision toward *recipient*).

Independent variable	*N* (clusters)	odds ratio±SE	*P*
DeSteno et al. [20] cues by *actor*	180 (90)	1.04±0.05	0.50
*Recipient*'s attractiveness	208 (104)	0.88±0.17	0.49
*Actor*'s zip code median income	200 (104)	1.04±0.14	0.78
*Actor*'s F1 LSRP^a^ score	208 (104)	1.17±0.16	0.27
*Recipient* interrupts *actor* (per min)	208 (104)	0.61±0.21	0.15
Interruption rate × *actor* F1 LSRP a	208 (104)	0.69±0.17	0.13
Common ground b × *actor* F1 LSRP a	208 (104)	1.26±0.25	0.25

1 = cooperate, 0 = defect.

a primary psychopathy.

b 1 = yes.

**Table 3:** Bivariate logistic regressions predicting 3^rd^ party insider guesses (*other* guessing *actor*'s PD decision toward *recipient*).

Independent variable	*N* (clusters)	odds ratio±SE	*P*
DeSteno et al. [20] cues by *actor*	180 (90)	0.99±0.06	0.90
*Recipient*'s attractiveness	210 (105)	1.39±0.24	0.06
*Actor*'s zip code median income	202 (105)	1.01±0.15	0.93
*Actor*'s F1 LSRP^a^ score	210 (105)	1.07±0.17	0.65
*Recipient* interrupts *actor* (per min)	210 (105)	0.65±0.23	0.22
Interruption rate × *actor* F1 LSRP a	210 (105)	0.79±0.22	0.39
Common ground b × *actor* F1 LSRP a	210 (105)	1.63±0.58	0.17

1 = cooperate, 0 = defect.

a primary psychopathy.

b 1 = yes.

**Table 4:** Bivariate logistic regressions predicting naïve video viewer guesses.

Independent variable	*N* (clusters)	odds ratio±SE	*P*
DeSteno et al. (20) cues by *actor*	356 (70)	1.08±0.05	0.11
*Recipient*'s attractiveness	416 (70)	1.03±0.09	0.75
*Actor*'s zip code median income	400 (70)	1.15±0.12	0.19
*Actor*'s F1 LSRP^a^ score	416 (70)	0.87±0.08	0.14
*Recipient* interrupts *actor* (per min)	416 (70)	0.92±0.16	0.63
Interruption rate × *actor* F1 LSRP^a^	416 (70)	0.77±0.14	0.15
Common ground^b^ × *actor* F1 LSRP^a^	416 (70)	0.74±0.14	0.12

1 = cooperate, 0 = defect.

a primary psychopathy.

b 1 = yes.

**Table 5:** Bivariate logistic regressions predicting informed video viewer guesses.

Independent variable	*N* (clusters)	odds ratio±SE	*P*
DeSteno et al. (20) cues by *actor*	359 (35)	1.00±0.04	0.95
*Recipient*'s attractiveness	419 (35)	1.09±0.11	0.41
*Actor*'s zip code median income	403 (35)	1.19±0.12	0.10
*Actor*'s F1 LSRP^a^ score	419 (35)	0.90±0.10	0.37
*Recipient* interrupts *actor* (per min)	419 (35)	1.17±0.30	0.54
Interruption rate × *actor* F1 LSRP^a^	419 (35)	0.62±0.08	<0.001
Common ground^b^ × *actor* F1 LSRP^a^	419 (35)	0.92±0.19	0.68

1 = cooperate, 0 = defect.

a primary psychopathy.

b 1 = yes.

**Table 6:** Multivariate logistic regression models predicting 2^nd^ party guesses (*recipient* guessing *actor*'s PD decision toward *recipient*).

Model	Model 1^a^	Model 2^b^	Model 3^c^	Model 4^d^
*N* (clusters)	196 (102)	200 (104)	171 (101)	174 (103)
Independent variable				
*Recipient*'*s* PD decision toward *actor*	112.42±69.29***	–	115.40±75.59***	–
DeSteno et al. [20] cues by *actor*	–	–	1.00±0.08	1.04±0.06
*Recipient*'s attractiveness	1.02±0.29	0.85±0.16	1.00±0.32	0.81±0.17
*Actor*'s zip code median income	0.98±0.21	0.98±0.14	1.00±0.25	1.00±0.17
*Actor*'s F1 LSRP^e^ score	1.71±0.47*	1.34±0.29	1.59±0.45	1.32±0.31
*Recipient* interrupts *actor* (per min)	1.54±0.76	0.58±0.22	1.34±0.74	0.67±0.28
*Actor*/*recipient* CG^f^	2.23±1.02§	1.83±0.60§	2.04±1.05	1.67±0.60
Interruption rate × *actor* F1 LSRP^e^	0.67±0.20	0.71±0.18	0.79±0.23	0.73±0.21
CG^f^ × *actor* F1 LSRP^e^	0.48±0.21	1.04±0.35	0.50±0.23	1.09±0.39
Wald χ^2^	65.49	8.64	61.89	6.06
r^2^	0.49	0.04	0.48	0.03
*P*	<0.0001	0.28	<0.0001	0.64

1 = cooperate, 0 = defect. §*P*<0.10. *P<0.05. ***P<0.001.

a Gervais et al. [45] predictor variables including *recipient*'s PD decision toward *actor.*

b Gervais et al. [45] predictor variables excluding *recipient*'s PD decision toward *actor*.

c Including DeSteno et al. [20] cues and *recipient*'s PD decision.

d Including DeSteno et al. [20] cues, excluding *recipient*'s PD decision.

e primary psychopathy.

f Common ground. 1 = yes.

**Table 7:** Multivariate logistic regression models predicting 3^rd^ party insider guesses (*other* guessing *actor*'s PD decision toward *recipient*).

Model	Model 1^a^	Model 2^b^	Model 3^c^	Model 4^d^
*N* (clusters)	200 (104)	202 (105)	174 (103)	174 (103)
Independent variable				
*Other*'s guess of *actor*'s PD decision toward *other*	90.08±56.21***	–	188.51±151.12***	–
DeSteno et al. [20] cues by *actor*	–	–	0.93±0.08	0.98±0.06
*Recipient*'s attractiveness	1.60±0.34*	1.30±0.24	1.76±0.45*	1.27±0.25
*Actor*'s zip code median income	0.96±0.18	1.00±0.15	1.03±0.22	1.04±0.17
*Actor*'s F1 LSRP^e^ score	0.88±0.32	1.08±0.25	0.73±0.27	1.03±0.25
*Recipient* interrupts *actor* (per min)	0.51±0.26	0.62±0.22	0.76±0.50	0.83±0.33
*Actor*/*recipient* CG^f^	1.32±0.64	1.85±0.75	1.18±0.70	1.70±0.74
Interruption rate × *actor* F1 LSRP^e^	1.21±0.58	0.72±0.25	1.33±0.63	0.76±0.27
CG^f^ × *actor* F1 LSRP^e^	1.81±1.08	1.82±0.65§	1.95±1.43	1.81±0.64§
Wald χ^2^	63.54	10.53	71.57	8.09
r^2^	0.50	0.05	0.55	0.04
*P*	<0.0001	0.16	<0.0001	0.42

1 = cooperate, 0 = defect. §*P*<0.10. *P<0.05. ***P<0.001.

a Gervais et al. (45) predictor variables including *other*'s guess of *actor*'*s* PD decision toward *other.*

b Gervais et al. (45) predictor variables excluding *other*'s guess of *actor*'*s* PD decision toward *other*.

c Including DeSteno et al. [20] cues and *other*'*s* guess of *actor*'*s* PD decision toward *other*.

d Including DeSteno et al. [20] cues, excluding *other*'*s* guess of *actor*'*s* PD decision toward *other.*

e primary psychopathy.

f Common ground. 1 = yes.

**Table 8:** Multivariate logistic regression models predicting video viewers' guesses of *actor*'s PD decision toward *recipient*.

	Naïve video viewers		Informed video viewers	
Model	Model 1^a^	Model 2^b^	Model 1^a^	Model 2^b^
*N* (clusters)	400 (70)	344 (70)	403 (35)	347 (35)
Independent variable				
DeSteno et al. [20] cues by *actor*	–	1.07±0.05	–	0.98±0.04
*Recipient*'s attractiveness	1.10±0.10	1.15±0.12	1.13±0.13	1.15±0.12
*Actor*'s zip code median income	1.17±0.13	1.08±0.13	1.22±0.13§	1.20±0.14
*Actor*'s F1 LSRP^c^ score	1.14±0.21	1.11±0.23	1.12±0.18	1.11±0.20
*Recipient* interrupts *actor* (per min)	1.17±0.23	1.22±0.29	1.58±0.42§	1.65±0.44§
*Actor*/*recipient* common ground^d^	0.77±0.13	0.69±0.14§	1.38±0.38	1.23±0.35
Interruption rate × *actor* F1 LSRP^c^	0.77±0.12	0.92±0.18	0.59±0.08***	0.63±0.10**
Common ground^d^ × *actor* F1 LSRP^c^	0.77±0.15	0.61±0.13*	1.00±0.21	1.00±0.23
Wald χ^2^	13.36	16.90	21.25	17.00
r^2^	0.02	0.03	0.03	0.02
*P*	0.06	0.03	0.003	0.03

1 = cooperate, 0 = defect. §*P*<0.10. *P<0.05. **P<0.01. ***P<0.001.

a Gervais et al. [45] predictor variables.

b Gervais et al. [45] predictor variables plus DeSteno et al. [20] cues by *actor.*

c primary psychopathy.

d 1 = yes.

### Bivariate Tests

In general, the bivariate tests (Tables 2, 3, 4, and 5) show that neither the DeSteno et al. [20] cues nor the Gervais et al. [48] independent variables performed at better than chance levels in predicting the guesses of any class of guesser. The exceptions were that 3^rd^ party insiders were marginally more likely to guess cooperation when the *recipient* was more attractive, and informed video viewers were significantly influenced toward greater accuracy by the interaction between *actor*'s primary psychopathy score and *recipient*'s rate of interrupting *actor*, i.e. they expected *actors* higher in primary psychopathy to be less likely to cooperate toward *recipients* who had interrupted them more frequently.

### Multivariate Models: Predictors of Second Party Guesses

Table 6 shows the results of four multivariate logistic regression models in which the outcome variable is *recipient*'s guess of *actor*'s PD decision toward him/herself (2^nd^ party guesses). Among *recipients*' guesses of *actors*' PD decisions toward themselves, 177/204 (86.8%) were concordant with the *recipient*'*s* own PD decision toward that *actor* (i.e. decisions to cooperate were accompanied by expectations of cooperation, and decisions to defect were accompanied by expectations of defection). Thus, it is unsurprising that *recipient*'s PD decision toward *actor* massively predicts *recipient*'s guess of *actor*'s PD decision toward *recipient*. Models 2 and 4, which lack this independent variable, perform quite poorly at predicting *recipient*'s guess of *actor*'s PD decision. Within the multivariate models, the DeSteno et al. [20] cues had no independent effect on *recipients*' guesses, nor did their inclusion improve the predictive power of the models. Without the DeSteno et al. [20] cues, and controlling for *recipient*'s PD decision toward *actor*, *recipients* significantly expected *actors* higher in primary psychopathy to be *more* likely to cooperate toward them.

### Multivariate Models: Predictors of Third Party Insider Guesses

Table 7 shows analogous models of predictors of *others*' guesses of *actors*' PD decisions toward *recipients* (3^rd^ party insider guesses). Here, the models vary with respect to their inclusion, among the independent variables, of (1) *others*' guesses of *actors*' PD decisions toward themselves and (2) *actors*' DeSteno et al. [20] cues. Given our finding that 75.2% of conversation participants predicted that both their co-participants would make the same PD decision toward both themselves and the other co-participants, it is unsurprising that *other*'s prediction of *actor*'s PD decision toward *recipient* was largely a function of *other*'s prediction of *actor*'s PD decision toward him- or herself. Controlling for the latter independent variable, *others* expected *actors* to be more likely to cooperate toward more attractive *recipients*. Within the multivariate models, the DeSteno et al. [20] cues had no independent effect on *others*' guesses, although they slightly improved the predictive power of the model that included, as an independent variable, *other*'s prediction of *actor*'s PD decision toward him- or herself.

### Multivariate Models: Predictors of Video Viewers' Guesses

Table 8 shows models of the Gervais et al. [48] independent variables, with and without the DeSteno et al. [20] cues, as predictors of video viewers' guesses (3^rd^ party outsider guesses). None of the models explained more than 3% of the variance in video viewers' guesses. Within the multivariate models, the DeSteno et al. [20] cues had no independent effect on video viewers' guesses. Naïve video viewers appeared to be influenced in their guesses by the interaction between *actor*'s primary psychopathy score and the discovery of *actor*-*recipient* common ground, but in the opposite direction of the actual effect on game play: naïve video viewers expected that following the discovery of common ground, *actors* higher in primary psychopathy would be *more* likely to defect. Informed video viewers were influenced in the correct direction by the interaction between *actor*'s primary psychopathy score and *recipient*'s interruptions of actor: they expected *actors* higher in primary psychopathy to be more likely to defect toward *recipients* that had interrupted them more frequently.

### No relation between DeSteno et al. [20] cues and actual PD decisions

Participants who displayed the DeSteno et al. [20] cues at higher rates were no more likely to defect than individuals who displayed them at lower rates. This was the case regardless of whether we used PD decisions as data points (*N* = 176, o.r.  = 0.97±0.07, *P*>0.6) or we used individuals as data points and carried out an ordered logistic regression with a three-level dependent variable (1 = defect toward both co-participants; 2 = mixed decision; 3 = cooperate toward both co-participants: *n*_1_ = 28, *n*_2_ = 6, *n*_3_ = 54, coefficient = −0.03, *P* = 0.67). The mean (±SD) unit-weighed DeSteno et al. [20] cue frequencies of these three classes of participants were, respectively, 5.09±3.25, 3.83±1.60, and 4.71±2.94.

When we added the DeSteno et al. [20] cues to the predictive model of PD decisions described in the Introduction [48], it had no significant independent relationship to PD play, and the proportion of variance explained by the model fell slightly.

## Discussion

In natural social life, judgments of others' propensities to cooperate occur under many different circumstances. Experimental work on defector-detection should seek to simulate a wide range of these contexts, to illuminate the scope and limits of whatever defector-detection mechanisms have evolved in humans. In this paper we add to existing literature by conducting a “small talk” session among participants, and only later introducing them to the prisoner's dilemma game. This resembles everyday first meetings between strangers who may later cooperate with each other, but still maintains experimental control by using an economic game. We coded conversational behavior, and used the game data, other self-report data and attractiveness ratings of participants to (1) assess the accuracy and the between-participant reliability of gameplay guesses and (2) document empirical predictors of the guesses themselves, and examine how these varied across types of judges. By comparing four categories of guessers – recipients of PD decisions, third party insiders, and naïve and informed outsiders – we accounted for the cognitive load of conversation participation, and for the importance of foreknowledge of the upcoming game.

### Insiders' guesses are not accurate

Conversation participants were no better than chance at predicting gameplay decisions. Our data suggest that this may be because participants base their predictions of an actor's play on their own decision toward that actor, and base third-party predictions largely on their second-party predictions for that actor. Although the first of these heuristics was erroneous, the second was generally valid—actors did tend to make the same decision toward both co-participants.

In this study, as in DeSteno et al. [20], participants made their own gameplay decisions before being asked to make predictions. As a result, participants may have aligned their predictions with their own PD decisions. In Reed et al. [19], participants predicted co-participants' PD decisions before making their own decisions; in Frank et al. [17] and Brosig [18], the order is not made explicit. Either cognitive dissonance reduction or false consensus beliefs may explain why actors failed to accurately anticipate defection. False consensus beliefs in social dilemma decisions refer to the tendencies of selfish individuals to believe that most people are selfish, while (to a lesser extent) prosocial individuals believe that most people are prosocial [36], [53].

We report elsewhere that socioeconomic status and subclinical psychopathy are predictive of players' choices to cooperate or defect [48]. Both socioeconomic status [54] and psychopathy [55] can be judged accurately from thin slices of behavior. Thus, our participants had the potential to use an implicit version of the Gervais et al. [48] model to discern others' likelihood of cooperation or defection, although we have no evidence that they did accurately judge SES or psychopathy.

We argue that participants are failing to predict defection because they are depending on a flawed folk model of defection, and not because they unable to detect relevant cues. Holding guessers' own PD decisions constant, we found that recipients were more likely to predict cooperation by co-participants who were *higher* in primary psychopathy. This supports the view [56] that psychopathy includes a convincingly “charming” self-presentation, and that subclinical primary psychopathy may support adaptive unilateral defection (see [48]). In general, third party insiders seem to expect that others will favor more attractive participants—they are more likely to predict cooperation by an actor towards more attractive participants (when controlling for how they expect that actor to play towards themselves). Since actors are more likely to cooperate toward more attractive co-participants, this can actually boost predictive accuracy.

### Naïve outsiders' guesses are only moderately accurate

Naïve video viewers, unlike conversation participants, approached better-than-chance prediction accuracy, and their cross-sex predictions were significantly more accurate than chance. The latter (unpredicted) result might reflect the operation of domain-specific mechanisms for detecting untrustworthiness in the opposite sex and thereby avoiding sexual exploitation (abandonment, cuckoldry, etc.). We are skeptical of this interpretation, because all conversation groups were single-sex—whereas such a domain-specific mechanism would likely require observation of between-sex interactions. Conversation participants rarely discussed romantic relationships, and almost all such discussion was brief and superficial. Moreover, ancestral humans presumably lived in groups in which intra-sexual cooperation and trustworthiness were comparable, in fitness-relevance, to intersexual cooperation and trustworthiness [57], [58]. An alternate — and, we think, more likely —interpretation is that naïve video viewers paid closer attention, on average, to video of opposite-sexed conversation groups, resulting in better perception of cues of trust and trustworthiness.

Naïve video viewers may be more accurate than conversation participants for several reasons. First, since video viewers made no PD decisions, they were free from the effects of cognitive dissonance reduction and false consensus beliefs. Video viewers were also free of the cognitive load entailed by the demands of self-presentation during a face-to-face interaction [47]. Finally, they were free of any neurophysiological or hormonal effects of face-to-face interactions [45], [46]. We did not directly measure any such effects. However, our finding that video viewers, unlike conversation participants, significantly underestimated overall rates of cooperation, is consistent with the view that distinctive characteristics of participating in (and not merely witnessing) face-to-face interaction elevate general expectations of prosociality, though they fail to improve, and may even worsen, predictive accuracy. Interestingly, Vogt et al. ([43]; C. Efferson, personal communication) found no significant differences between overall rates of guesses of cooperation and overall rates of actual cooperation when the stimuli were videotaped monologues rather than videotaped conversations.

Video viewers experienced the disadvantage of viewing only one person *en face*. Since facial expressions (particularly Duchenne smiles) are important for cooperator-detection [19], [24], [25], [27], [59], video-viewers should be most accurate in their predictions about the single *en face* player. This prediction received no support from our data, but perhaps video viewers' predictions were based on dyad-level rather than individual-level cues. This interpretation is consistent with our findings that video viewers were more likely than conversation participants to predict individually divergent PD decisions.

The cues used by naïve video viewers in making their marginally accurate guesses remain a puzzle. They were not using a folk or implicit equivalent of the Gervais et al. [48] model of PD decision making, since the strongest effect from this model (the common ground-*actor* primary psychopathy interaction) was significant in the opposite direction of this variable's effect on actual PD decisions, and the main effect of *recipient*'*s* interruptions of *actor* also trended in the opposite direction to that observed on PD decisions. Nor were they using the De Steno et al. [20] cues.

### Informed outsiders' guesses are not accurate

Informed video viewers, who were presumably consciously seeking cues to post-conversation defection, failed to guess PD decisions at better-than-chance levels, and did not show the cross-sex effect found in naïve video viewers. These results are consistent with Bonnefon et al.'s [60] finding that people's accuracy at predicting behavior in a trust game declined with the availability of additional information. In predicting social dilemma decisions, more information may hurt rather than help. On the other hand, Vogt et al. [43] found that adding audio content to video of brief monologues increased (though nonsignificantly) viewers' accuracy at predicting anonymous PD decisions.

Our results may be explained by the confluence of three attributes of the informed video viewer condition, which together impeded accurate social judgment: (1) the behavioral slices (10-min triadic conversations) were “thick” enough to provide input into a wide variety of social judgment heuristics, the relative weight of which varied among video viewers; (2) conversation participants were ignorant of the post-conversation PD and were therefore not deliberately displaying or eliciting signs of prosociality or trustworthiness; and (3) uniquely to the informed video viewer condition, video viewers were seeking such signs, and they varied in the extent to which they processed the difference between their own perspective (knowing about the post-conversation PD) and the uninformed perspective of the conversation participants. In other words, the perspective-taking task required of the informed video viewers may have been too demanding for participants to yield accurate judgments. Informed video viewers needed to (1) distinguish between their own knowledge and the conversation participants' ignorance of the PD and (2) if they viewed the PD as an assurance game [32]–[34], judge the conversation participants' judgments of one another's trustworthiness.

Future research could determine whether individual variation in measures of social intelligence such as interpersonal sensitivity [61] is associated with accurate judgment in this or a similar task. The informed video viewer task was also, arguably, less ecologically valid than the naïve video viewers' task. Quick and automatic person perception along general dimensions (e.g., warmth and competence [62]) is a widely demonstrated process with obvious adaptive benefits. The more complex task that we asked of the informed video viewers may lie outside the range of problems that human social judgment mechanisms were designed to solve [63]. For reasons discussed above, viewing a social interaction among strangers while attending to signs of cooperative intent may be too contrived a situation to elicit accurate social judgments.

### Putative cues of defection were neither used by guessers nor predictive of game play

None of three independent sets of observers (conversation participants, naïve video viewers, or informed video viewers) used the unit-weighted average of *actors*' frequencies of *arms crossed*, *lean back*, *hand touch*, and *face touch* as a cue of probability of defecting. Nor was actual defection associated with a higher frequency of engaging in this set of behaviors. We tentatively suggest four reasons for this failure to replicate the result of DeSteno et al. [20]. First, their experimental protocol involved dyads, whereas ours involved conversational triads followed by dyadic games. As implied by DeSteno et al's [20] emphasis on the context-dependent nature of cues to untrustworthiness, the four aforementioned behaviors may cue dyadic disengagement rather than a stable individual propensity to defect. In a triadic interaction, many nonverbal behaviors (particularly self-directed behaviors) are not directed specifically toward either co-participant, and this may reduce their power to predict particular dyadic choices. In conjunction with our participants' strong tendencies to make consistent decisions toward both co-participants (a pattern probably generated by causes outside the conversation itself), this ambiguity could eliminate the cue validity of the DeSteno et al. [20] behaviors. Second, DeSteno et al. [20] used a social dilemma with five choices, whereas we used a standard PD, which provides only 2 choices. Thus, our study's measure of trustworthiness may have been insufficiently sensitive to detect the effect that they found. However, Tables 2, 3, 4, 5, 6, 7, and 8 show that we failed to observe even a consistent trend toward an association of these cues with defection. Third, although DeSteno et al. [20] write that “[t]here was no expectation that partners would see each other again (1551),” all their participants were drawn from the same undergraduate participant pool, and, unlike in our study, the experimenters made no effort to conceal partners' game-play choices from each other. Therefore, their study design obscures the distinction between trustworthiness and concern about reputation. Fourth, the four cues that predicted defection in DeSteno et al.'s [20] study were determined empirically from a set of 12 cues, and there is no compelling theoretical explanation for why these four, and no others, significantly predicted low offers. Thus, their result might not be generalizable to other samples or experimental designs.

## Conclusions

Our results have implications for the study of the evolution of human cooperation. Naïve outsiders, but not interaction participants, were able to accurately judge inclinations to cooperate or defect, although it is likely that the observed levels of accuracy would be insufficient to support the evolution of one-shot cooperation under realistic assumptions about the benefit-to-cost ratio of cooperation [43]. This suggests that honest signaling of intentions and accurate defector detection have played a limited role in how human prosociality evolved. This inference is consistent with a considerable body of theoretical literature, which indicates that on an evolutionary timescale such “greenbeard” signals of cooperative intent are easily eroded by cheaters, or deceptive defectors who signal intent to cooperate [13], [14], [64]–[66]. Alternative theoretical explanations for human cooperation in one-shot social dilemmas, such as cultural group selection [2], [7] or the ancestral ubiquity of repeated interactions [8], [67] may be more promising. In addition, our findings suggest that eavesdropping could be an important means of information gathering about potential social partners. Outstanding questions include (1) under what contexts social cognition is more efficient in eavesdroppers than in interlocutors, and (2) whether there are adaptive explanations for these differences.

## References

[1] Fehr E, Fischbacher U, Gächter S (2002) Strong reciprocity, human cooperation and the enforcement of social norms. Human Nature 13: 1–25 10.1007/s12110-002-1012-7 26192593

[2] Fehr E, Henrich J (2003) Is strong reciprocity a maladaptation? On the evolutionary foundations of human altruism. CSEifo Working Paper Series No. 859. doi:10.2139/ssrn.382950.

[3] Henrich J, Boyd R, Bowles S, Camerer CF, Fehr E, et al (2005) “Economic Man” in cross-cultural perspective: behavioral experiments in 15 small-scale societies. Behav Brain Sci 28: 795–855 10.1017/S0140525X05000142 16372952

[4] Hamilton WD (1964) The genetical evolution of social behavior. J Theor Biol 7: 1–51 10.1016/0022-5193(64)90039-6 5875341

[5] Williams GC, Williams DC (1957) Natural selection of individually harmful social adaptations among sibs with special reference to social insects. Evolution 11: 32–39.

[6] Trivers RL (1971) The evolution of reciprocal altruism. Q Rev Biol 46: 35–57.

[7] Boyd R, Gintis H, Bowles S, Richerson J (2003) The evolution of altruistic punishment. Proc Natl Acad Sci U S A 100: 3531–3535 10.1073/pnas.0630443100 12631700 PMC152327

[8] Krasnow M, Delton AW, Tooby J, Cosmides L (2013) Meeting now suggests we will meet again: implications for debates on the evolution of cooperation. Scientific Reports 3: 1747 10.1038/srep01747 23624437 PMC3638167

[9] Frank RH (1987) If Homo economicus could choose his own utility function, would he want one with a conscience? Am Econ Rev 77: 593–604.

[10] Frank RH (1988) Passions within Reason: The Strategic Role of the Emotions. New York: Norton.

[11] Hirshleifer J (1987) On the emotions as guarantors of threats and promises. In: Dupre J, editor. The Latests on the Best: Essays on Evolution and Optimality. Cambridge, MA: MIT Press. pp. 307–326.

[12] Dawkins R (1976) The Selfish Gene. Oxford: Oxford University Press.

[13] Efferson C, Vogt S (2013) Viewing men's faces does not lead to accurate predictions of trustworthiness. Scientific Reports 3: 1047 10.1038/srep01047 23308340 PMC3541508

[14] Henrich J (2004) Cultural group selection, coevolutionary processes and large-scale cooperation. Journal of Economic Behavior and Organization 53: 3–35 10.1016/S0167-2681(03)00094-5

[15] Fehr E, Fischbacher U (2005) Altruists with green beards. Analyse & Kritik 27: 73–84.

[16] Frank RH (2005) Altruists with green beards: still kicking? Analyse & Kritik 27: 85–96.

[17] Frank RH, Gilovich T, Regan DT (1993) The evolution of one-shot cooperation: an experiment. Ethology and Sociobiology 14: 247–256 10.1016/0162-3095(93)90020-I

[18] Brosig J (2002) Identifying cooperative behavior: some experimental results in a prisoner's dilemma game. Journal of Economic Behavior & Organization 47: 275–290 10.1016/S0167-2681(01)00211-6

[19] Reed LI, Zeglen KN, Schmidt KL (2012) Facial expressions as honest signals of cooperative intent in a one-shot anonymous Prisoner's Dilemma game. Evolution and Human Behavior 33: 200–209 10.1016/j.evolhumbehav.2011.09.003

[20] DeSteno D, Breazeal C, Frank RH, Pizarro D, Baumann J, et al (2012) Detecting the trustworthiness of novel partners in economic exchange. Psychological Science 23: 1549–1556 10.1177/0956797612448793 23129062

[21] Yamagishi T, Kikuchi M, Kosugi M (1999) Trust, gullibility, and social intelligence. Asian Journal of Social Psychology 2: 145–161 10.1111/1467-839X.00030

[22] Brown WM, Palameta B, Moore C (2003) Are there nonverbal cues to commitment? An exploratory study using the zero-acquaintance video presentation paradigm. Evolutionary Psychology 1: 42–69.

[23] Centorrino S, Djemai E, Hopfensitz A, Milinski M, Seabright P (2011) Smiling is a costly signal of cooperation opportunities: experimental evidence from a trust game. IDEI Working Paper 669..

[24] Mehu M, Grammar K, Dunbar RIM (2007) Smiles when sharing. Evolution and Human Behavior 28: 415–422 10.1016/j.evolhumbehav.2007.05.010

[25] Oda R, Yamagata N, Yabiku Y, Matsumoto-Oda A (2009) Altruism can be assessed correctly based on impression. Human Nature 20: 331–341 10.1007/s12110-009-9070-8

[26] Boone RT, Buck R (2003) Emotional expressivity and trustworthiness: the role of nonverbal behavior in the evolution of cooperation. Journal of Nonverbal Behavior 27: 163–182 10.1023/A:1025341931128

[27] Schug J, Matsumoto D, Horita Y, Yamagishi T, Bonnet K (2010) Emotional expressivity as a signal of cooperation. Evolution and Human Behavior 31: 87–94 10.1016/j.evolhumbehav.2009.09.006

[28] Fetchenhauer D, Groothuis T, Pradel J (2010) Not only states but traits - humans can identify permanent altruistic dispositions in 20 s. Evolution and Human Behavior 31: 80–86 10.1016/j.evolhumbehav.2009.06.009

[29] Little AC, Jones BC, DeBruine LM, Dunbar RIM (2013) Accuracy in discrimination of self-reported cooperators using static facial information. Personality and Individual Differences 54: 507–512 10.1016/j.paid.2012.10.018

[30] Stirrat M, Perrett DI (2010) Valid facial cues to cooperation and trust: male facial width and trustworthiness. Psychological Science 21: 349–354 10.1177/0956797610362647 20424067

[31] De Neys W, Hopfensitz A, Bonnefon J-F (2013) Low second-to-fourth digit ratio predicts indiscriminate social suspicion, not improved trustworthiness detection. Biology Letters 9: 20130037 10.1098/rsbl.2013.0037 23445949 PMC3639781

[32] Fehr E, Camerer CF (2007) Social neuroeconomics: the neural circuitry of social preferences. Trends in Cognitive Sciences 11: 419–427 10.1016/j.tics.2007.09.002 17913566

[33] Hayashi N, Ostrom E, Walker J, Yamagishi T (1999) Reciprocity, trust, and the sense of control: a cross-societal study. Rationality and Society 11: 27–46 10.1177/104346399011001002

[34] Kiyonari T, Tanida S, Yamagishi T (2000) Social exchange and reciprocity: confusion or a heuristic? Evolution and Human Behavior 21: 411–427 10.1016/S1090-5138(00)00055-6 11146306

[35] Kurzban R, Houser D (2005) Experiments investigating cooperative types in humans: a complement to evolutionary theory and simulations. Proceedings of the National Academy of Sciences 102: 1803–1807 10.1073/pnas.0408759102 PMC547861 15665099

[36] Bogaert S, Boone C, Declerck C (2008) Social value orientation and cooperation in social dilemmas: a review and conceptual model. Br J Soc Psychol 47: 453–480 10.1348/014466607X244970 17915044

[37] Ambady N, Rosenthal R (1992) Thin slices of expressive behavior as predictors of interpersonal consequences - a metaanalysis. Psychol Bull 111: 256–274 10.1037/0033-2909.111.2.256

[38] Vanneste S, Verplaetse J, Van Hiel A, Braeckman J (2007) Attention bias toward noncooperative people. A dot probe classification study in cheating detection. Evolution and Human Behavior 28: 272–276 10.1016/j.evolhumbehav.2007.02.005

[39] Dawes RM, McTavish J, Shaklee H (1977) Behavior, communication, and assumptions about other people's behavior in a commons dilemma situation. J Pers Soc Psychol 35: 1–11 10.1037/0022-3514.35.1.1

[40] De Paulo BM, Lindsay JJ, Malone BE, Muhlenbruck L, Charlton K, et al (2003) Cues to deception. Psychol Bull 129: 74–118 10.1037/0033-2909.129.1.74 12555795

[41] Ambady N, Bernieri FJ, Richeson JA (2000) Toward a histology of social behavior: Judgmental accuracy from thin slices of the behavioral stream. In: Zanna MP, editor. Advances in Experimental Social Psychology. San Diego: Academic Press. pp. 201–271.

[42] Verplaetse J, Vanneste S, Braeckman J (2007) You can judge a book by its cover: the sequel. A kernel of truth in predictive cheating detection. Evolution and Human Behavior 28: 260–271 10.1016/S1090-5138(03)00035-7

[43] Vogt S, Efferson C, Fehr E (2013) Can we see inside? Predicting strategic behavior given limited information. Evolution and Human Behavior. doi:10.1016/j.evolhumbehav.2013.03.003.

[44] Yamagishi T, Tanida S, Mashima R, Shimoma E, Kanazawa S (2003) You can judge a book by its cover: evidence that cheaters may look different from cooperators. Evolution and Human Behavior 24: 290–301 10.1016/S1090-5138(03)00035-7

[45] Rilling JK, Gutman DA, Zeh TR, Pagnoni G, Berns GS, et al (2002) A neural basis for social cooperation. Neuron 35: 395–405 10.1016/S0896-6273(02)00755-9 12160756

[46] Zak PJ, Kurzban R, Matzner WT (2005) Oxytocin is associated with human trustworthiness. Horm Behav 48: 522–527 10.1016/j.yhbeh.2005.07.009 16109416

[47] Pontari BA, Schlenker BR (2000) The influence of cognitive load on self-presentation: can cognitive busyness help as well as harm social performance? J Pers Soc Psychol 78: 1092–1108 10.1037/0022-3514.78.6.1092 10870911

[48] Gervais MM, Kline M, Ludmer M, George R, Manson JH (2013) The strategy of psychopathy: primary psychopathic traits predict defection on low-value relationships. Proc R Soc Lond B Biol Sci 280: 20122773 10.1098/rspb.2012.2773 PMC3619474 23446522

[49] Levenson MR, Kiehl KA, Fitzpatrick CM (1995) Assessing psychopathic attributes in a noninstitutionalized population. J Pers Soc Psychol 68: 151–158 10.1037/0022-3514.68.1.151 7861311

[50] Fischbacher U (2007) z-Tree: Zurich toolbox for ready-made economic experiments. Experimental Economics 10: 171–178 10.1007/s10683-006-9159-4

[51] von Eye A, Mun EY (2005) Analyzing Rater Agreement: Manifest Variable Methods. Mahwah, NJ: Lawrence Erlbaum.

[52] Manson JH, Gervais MM, Kline M (2013) Data from: defectors cannot be detected during “small talk” with strangers. Dryad Digital Repository. doi:10.5061/dryad.qm689. 10.1371/journal.pone.0082531 PMC3865023 24358201

[53] Smeesters D, Warlop L, Van Avermaet E, Corneille O, Yzerbyt V (2003) Do not prime hawks with doves: the interplay of construct activation and consistency of social value orientation on cooperative behaviour. J Pers Soc Psychol 84: 972–987 10.1037/0022-3514.84.5.972 12757142

[54] Kraus MW, Keltner D (2009) Signs of socioeconomic status: a thin-slicing approach. Psychological Science 20: 99–106 10.1111/j.1467-9280.2008.02251.x 19076316

[55] Fowler KA, Lilienfeld SO, Patrick CJ (2009) Detecting psychopathy from thin slices of behavior. Psychological Assessment 21: 68–78 10.1037/a0014938 19290767

[56] Cleckley H (1988 (1941)) The Mask of Sanity. St. Louis, MO: C.V. Mosby.

[57] Hill K, Barton M, Hurtado AM (2009) The emergence of human uniqueness: characters underlying behavioral modernity. Evolutionary Anthropology 18: 187–200 10.1002/evan.20224

[58] Kaplan H, Hooper PL, Gurven M (2009) The evolutionary and ecological roots of human social organization. Philos Trans R Soc Lond B Biol Sci 364: 3289–3299 10.1098/rstb.2009.0115 19805435 PMC2781874

[59] Mehu M, Little AC, Dunbar RIM (2007) Duchenne smiles and the perception of generosity and sociability in faces. Journal of Evolutionary Psychology 5: 133–146 10.1556/JEP.2007.1011

[60] Bonnefon J-F, Hopfensitz A, De Neys W (2012) The modular nature of trustworthiness detection. J Exp Psychol. doi:1037/a0028930. 10.1037/a0028930 22686638

[61] Hall JA, Andrzejewski SA, Yopchick JE (2009) Psychosocial correlates of interpersonal sensitivity: a meta-analysis. Journal of Nonverbal Behavior 22: 149–180 10.1007/s10919-009-0070-5

[62] Fiske ST, Cuddy AJC, Glick P (2007) Universal dimensions of social cognition: warmth and competence. Trends in Cognitive Sciences 11: 77–83 10.1016/j.tics.2006.11.005 17188552

[63] Haselton MG, Funder D (2006) The evolution of accuracy and bias in social judgment. In: Schaller M, Kenrick DT, Simpson JA, editors. Evolution and Social Psychology.New York: Psychology Press. pp. 15–37.

[64] Dawkins R (1982) The Extended Phenotype. Oxford: Oxford University Press.

[65] Henrich J, Henrich N (2006) Culture, evolution and the puzzle of human cooperation. Cognitive Systems Research 7: 220–245 10.1016/j.cogsys.2005.11.010

[66] Price ME (2006) Monitoring, reputation, and “greenbeard” reciprocity in a Shuar work team. Journal of Organizational Behavior 27: 201–219 10.1002/job.347

[67] Delton AW, Krasnow M, Cosmides L, Tooby J (2011) Evolution of direct reciprocity under uncertainty can explain human generosity in one-shot encounters. Proceedings of the National Academy of Sciences 108: 13335–13340 10.1073/pnas.1102131108 PMC3156224 21788489

